# Rapid assessment of data systems for COVID-19 vaccination in the WHO African Region

**DOI:** 10.1017/S0950268824000451

**Published:** 2024-03-18

**Authors:** Franck Mboussou, Patrick Nkamedjie, Daniel Oyaole, Bridget Farham, Ajiri Atagbaza, Sheillah Nsasiirwe, Ana Costache, Donald Brooks, Charles Shey Wiysonge, Benido Impouma

**Affiliations:** 1 World Health Organization, Regional Office for Africa, Brazzaville, Congo; 2 JSI, Momentum, Arlington, VA, USA; 3 World Health Organization, Department of Immunization, Vaccines & Biologicals, Geneva, Switzerland

**Keywords:** African region, COVID-19 vaccines, digital solutions, DHIS2, data systems

## Abstract

Most countries in Africa deployed digital solutions to monitor progress in rolling out COVID-19 vaccines. A rapid assessment of existing data systems for COVID-19 vaccines in the African region was conducted between May and July 2022, in 23 countries. Data were collected through interviews with key informants, identified among senior staff within Ministries of Health, using a semi-structured electronic questionnaire. At vaccination sites, individual data were collected in paper-based registers in five countries (21.7%), in an electronic registry in two countries (8.7%), and in the remaining 16 countries (69.6%) using a combination of paper-based and electronic registries. Of the 18 countries using client-based digital registries, 11 (61%) deployed the District Health Information System 2 Tracker, and seven (39%), a locally developed platform. The mean percentage of individual data transcribed in the electronic registries was 61% ± 36% standard deviation. Unreliable Internet coverage (100% of countries), non-payment of data clerks’ incentives (89%), and lack of electronic devices (89%) were the main reasons for the suboptimal functioning of digital systems quoted by key informants. It is critical for investments made and experience acquired in deploying electronic platforms for COVID-19 vaccines to be leveraged to strengthen routine immunization data management.

## Key results

- Individual data at coronavirus disease 2019 (COVID-19) vaccine sites were collected in paper-based registers in five countries (21.7%) and in an electronic registry in two countries (8.7%), while 16 countries (69.6%) were using a combination of paper-based and digital registries.
- Of the 18 out of the 23 countries using client-based electronic registries, 11 (61%) deployed the District Health Information System 2 (DHIS2) Tracker, and seven (39%), a locally developed platform.
- The mean percentage of individual data transcribed in the electronic registries was 61% ± 36% standard deviation.
- Unreliable Internet coverage (100% of countries), non-payment of data clerks’ incentives (89%), and lack of electronic devices (89%) were the main reasons for the suboptimal functioning of electronic systems quoted by key informants.

## Introduction

The African region is one of the six regions of the World Health Organization (WHO) and is made up of 47 [1] out of the 54 countries of the African continent. Since February 2020, the African region has been dealing with the coronavirus disease 2019 (COVID-19) pandemic and its socioeconomic impact, even though the public health emergency of international concern status was lifted in May 2023 [2]. Early responses included the implementation of public health and social measures of varying intensities and durations to interrupt community transmission [3]. The accelerated development and approval of COVID-19 vaccines for emergency use led to vaccination being added to the response interventions less than one year after the onset of the COVID-19 pandemic [4, 5]. In October 2021, the WHO published an ambitious strategy to ensure that all countries including African countries had vaccinated 40% of their population by the end of 2021 and 70% by mid-2022 [6]. The goal was to substantially increase population immunity globally, prevent hospitalizations and thus protect health systems, prevent deaths, fully restart economies, and lower the risk of new variants [6]. The WHO recommended that countries focus first on protecting health workers and other essential workers, older adults, and people at high risk of severe disease and death because of comorbidities, before advancing to all adults and adolescents [6].

To help countries monitor progress in rolling out COVID-19 vaccines, the WHO developed and disseminated an interim guidance for monitoring COVID-19 vaccines [7]. This guidance document outlined considerations for the collection and use of vaccination data and provided templates for paper-based data collection tools including registers, tally sheets, reporting forms, and vaccination cards [7]. In addition to paper-based tools for COVID-19 vaccine data collection, some countries have deployed the District Health Information System 2 (DHIS2) and/or other digital solutions to collect individual or aggregate data on vaccine uptake. Over one year into the COVID-19 vaccine rollout, the WHO African Regional Office for Africa (WHO AFRO) was still struggling with the timeliness and completeness of the data reported by its member states, especially on vaccination coverage for high-priority groups (health workers, older adults, and people with comorbidities). This was partially due to suboptimal data management systems for COVID-19 vaccines, including in countries that deployed digital solutions.

To identify gaps in COVID-19 vaccine data management in countries in the African region, the WHO AFRO conducted, between May and July 2022, a rapid assessment of existing data systems for COVID-19 vaccines in the African region. This paper summarizes the key findings of the assessment and discusses its implications for routine immunization.

## Methods

A descriptive cross-sectional study was conducted in the WHO African Region to assess the existing data systems for COVID-19 vaccines, from 5 May 2022 to 15 July 2022.

### Inclusion and exclusion criteria

Countries belonging to the African region that had started to roll out COVID-19 vaccines and that agreed to participate in the survey by responding to the WHO AFRO request of key informants were included.

The only country in the region that had not yet started to implement COVID-19 vaccines by then (Eritrea) was excluded.

Among the 46 countries that were rolling out COVID-19 vaccines, 23 agreed to participate in this survey.

### Data collection

Data were collected through interviews with key informants from the Ministries of Health (MoH), using a semi-structured questionnaire designed on the Open Data Kit (ODK) [8]. The WHO country offices identified key informants among senior staff within the MoH in charge of COVID-19 vaccine data management. Following the formal nomination of key informants, online meetings were set up to carry out data collection interviews. Each key informant was interviewed individually by investigators from the WHO AFRO. The following information was collected: the existing platform or system for data collection, the type of data collected by the electronic platform if applicable (individual or aggregated), the number of vaccination sites using the existing platform, the percentage of transcribed client records entered into the electronic platform, the availability of an offline data recording option, the existence of a parallel system for collecting aggregate data, means of transmission of aggregate data across the health system, the use of data from the electronic platform to feed information products (such as situation reports, bulletins, or PowerPoint presentation on COVID-19 vaccines) and inform decisions, reasons for possible suboptimal functioning of the data systems, and the issuance of digital vaccination certificates.

### Data analysis

Data were analysed using R version 4.2.1 [9] for statistical analysis. ESRI 2017 ArcGIS Pro 2.1.0 [10] was used to generate maps. Content analysis was performed for open questions such as ‘reasons for possible suboptimal functioning of the data systems’.

## Results

### Study population (participating countries)

Twenty-three countries participated in this survey including eight from Western Africa (Burkina Faso, Côte d’Ivoire, Gambia, Ghana, Guinea, Niger, Senegal, and Sierra Leone), six from Central Africa (Cameroon, Central African Republic (CAR), Chad, Congo, Democratic Republic of the Congo (DRC), and Gabon), five from Eastern Africa (Burundi, Kenya, South Sudan, Tanzania, and Uganda), and four from Southern Africa (Eswatini, Lesotho, Malawi, and Zambia).

### Platforms for COVID-19 data collection, recording, and reporting at vaccination sites

Five countries (21.7%) were using only paper-based registers for individual data collection at vaccination sites (Chad, South Sudan, CAR, Niger, and Senegal), including four countries using, additionally, an electronic platform to collect aggregate data (ODK for South Sudan, DHIS2 for Senegal, HIS for Chad, and Portail de vaccination for Niger). CAR was using an Excel sheet to collect aggregate data from districts.

Two countries (8.7%) were using an electronic platform for individual data collection at vaccination sites, exclusively (Eswatini and Burundi). The remaining 16 countries (69.6%) were using a combination of paper-based registers and electronic platforms for data collection: Burkina Faso, Cameroon, Congo, Côte d’Ivoire, DRC, Gabon, Gambia, Ghana, Guinea, Kenya, Lesotho, Malawi, Sierra Leone, Tanzania, Uganda, and Zambia. These countries stated that they have kept the paper-based reporting system in place despite having deployed an electronic platform, because they anticipated that not all data would be recorded in the electronic platform. Among the 16 countries, 12 had set up a parallel system for collecting aggregate data and four were not collecting aggregate data (Gabon, Kenya, Uganda, and Zambia).

**Figure 1.**
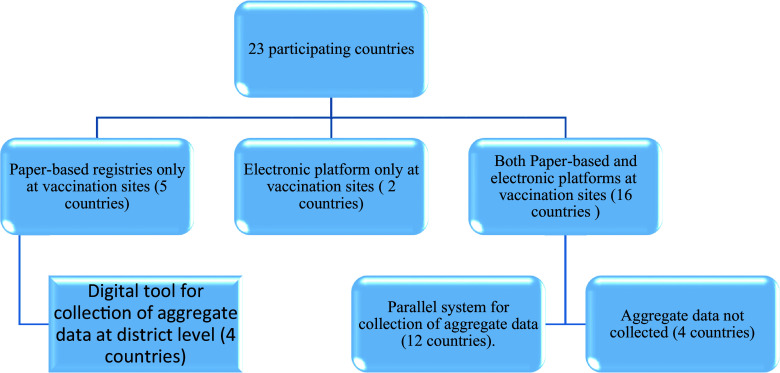
Diagram of data systems for COVID-19 vaccines in 23 countries in the African region (data as of 15 July 2022).

Uganda was using Smart Paper Technology to scan data from paper registers and upload them into DHIS2. Figure 1 summarizes the situation of participating countries according to existing data systems for COVID-19 vaccines. Overall, 22 out of the 23 (96%) countries deployed an electronic platform to collect COVID-19 vaccine data, including 18 (78%) with a client-based tool (electronic immunization registries) and four (22%) collecting only aggregate data.

Of the 18 countries using an electronic platform to collect individual data at vaccination sites, 11 (61%) were using the DHIS2 Tracker. These were Burkina Faso, Cameroon, DRC, Gambia, Ghana, Guinea, Lesotho, Malawi, Sierra Leone, Uganda, and Zambia. The electronic platform for individual data collection deployed in the remaining seven countries was Chanjo-Ke in Kenya (Chanjo means vaccination in Swahili), CovaxPortal (Chanjo COVID in Swahili) in Tanzania, the COVID-19 platform developed by SaH Analytics in Cote d’Ivoire, SIARP (Integrated Information System for Early Warning and Response) in Burundi, SIRCOV (Integrated System for COVID-19 Response) in Congo, Research Electronic Data Capture (REDcap) in Gabon, and Eswatini COVID-19 vaccine (ESWAVAX) digital platform in Eswatini. Of the seven countries not using the DHIS2 Tracker for individual data collection, an offline record option was available only in two countries (Congo and Eswatini). In Kenya, Cote d’Ivoire, Burundi, Gabon, and Tanzania, availabilty of Internet was required to access the platform and record data with real-time synchronization.

**Figure 2.**
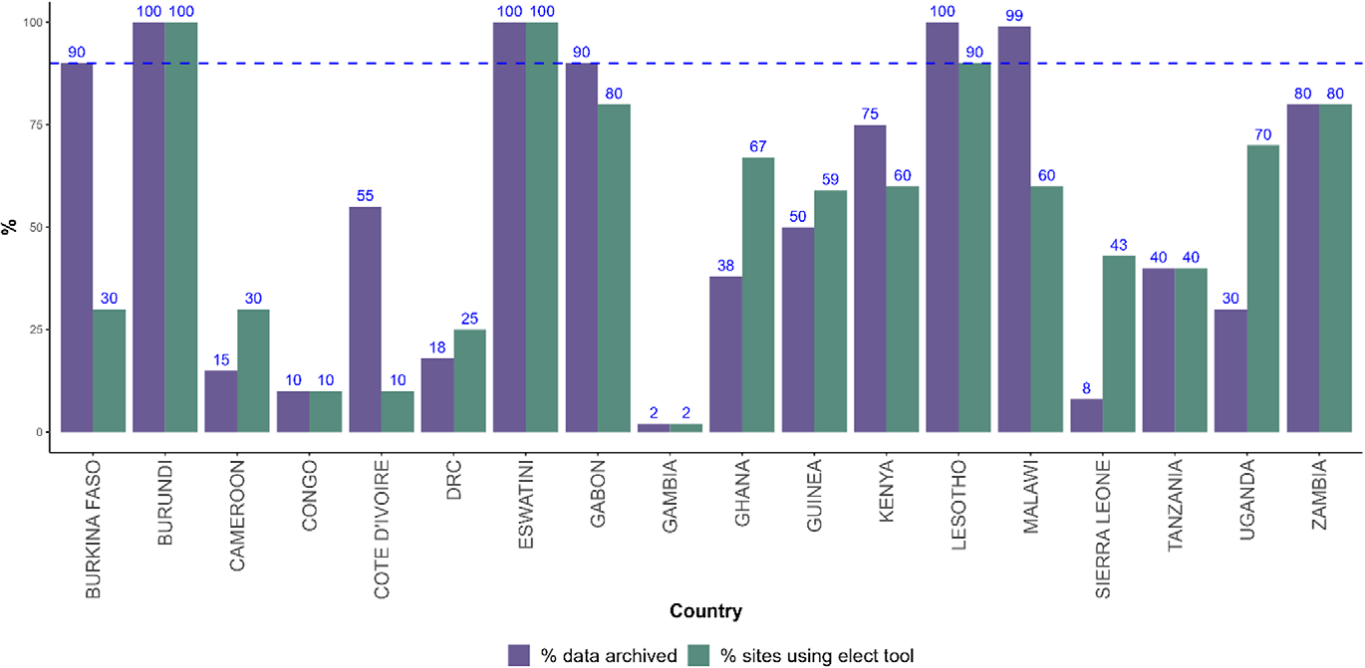
Percentage of vaccination sites utilizing the electronic platform for COVID-19 data collection and percentage of individual data captured. Data from 18 countries in the WHO African Region (data as of 15 July 2022).

Figure 2 presents the distribution of electronic platforms used for individual data collection in 23 countries.

### Data completeness from electronic platforms

**Figure 3.**
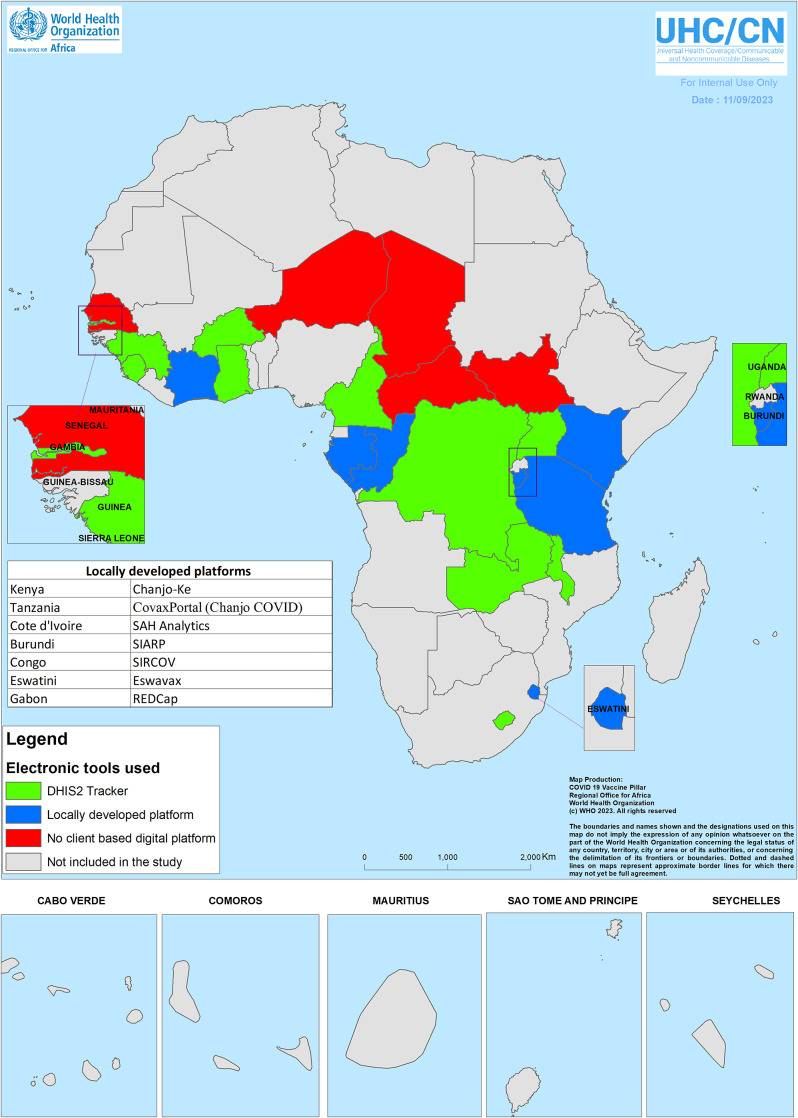
Client-based digital tool for COVID-19 vaccines deployed in 23 countries in the African region.

Among the 18 countries that were using electronic platforms to collect individual data, the mean percentage of fixed vaccination sites using these electronic platforms was 61% ± 36% standard deviation (range: 2%; 100%). In Gambia, the electronic platform (DHIS2 Tracker) was piloted in two out of the 82 (2%) service points; only people who had to travel were recorded to generate digital certificates. Burundi and Eswatini were the only countries with 100% vaccination sites using the electronic platform (Figure 3). Burundi had only six vaccination fixed sites including four in the capital city, while Eswatini had as many as 14 vaccination fixed sites. The mean percentage of fixed vaccination sites using these electronic platforms was significantly higher in countries not using the DHIS2 Tracker (82%) than in those using the DHIS2 Tracker (48%) (p = 0.04).

The mean percentage of individual’ data transcribed into the electronic platform among all 18 countries was 58% % ± 30% standard deviation (range: 2%; 100%). Eswatini and Burundi were the only countries with 100% of individual data transcribed. Uganda recorded 70% of data while having only 30% of vaccination sites using the electronic platform, as a result of the use of Smart Paper Technology. Gabon, Zambia, and Kenya were the only countries with less than 100% of individual data transcribed, using data from the electronic platform to feed information products. The mean percentage of individual data entered in the digital platform was 70% in countries not using the DHIS2 Tracker versus 50% in countries using the DHIS2 Tracker (difference not significant, p = 0.24).

Among the remaining 13 countries using an electronic registry, 12 set up parallel mechanisms for collecting aggregate data to be used to monitor progress and feed information products, except for Uganda. Of these, two (15%) countries (Cameroon and Côte d’Ivoire) were using DHIS2, and 10 countries (85%), an Excel template (Burkina Faso, Congo, DRC, Gambia, Ghana, Guinea, Lesotho, Malawi, Sierra Leone, and Tanzania).

Key informants identified the following reasons for the suboptimal functioning of digital systems for COVID-19 vaccines: unreliable Internet coverage especially in rural areas (100% of key informants), non-payment of data clerks’ incentives (quoted by 89%), lack of electronic devices (89%), insufficient number of data clerks (89%), limited skills of vaccination teams on data management (78%), non-provision of Internet bundles (77%), lack of supervision (55%), and high turnover of staff (44%).

### Recording of the COVID-19 vaccine history data of new COVID-19 cases admitted to health facilities

Twelve out of the 23 countries stated that the COVID-19 vaccine status was consistently and systematically recorded for new COVID-19 cases admitted to health facilities (52%). Of these 12 countries, nine stated that these data were not systematically shared with the COVID-19 vaccine rollout teams for their use in risk communication. Only three countries (Kenya, Malawi, and Tanzania) stated that an information-sharing mechanism between COVID-19 case management and vaccine rollout teams was set up.

### Provision of COVID-19 vaccine certificates after vaccination

In all 18 countries using an individual client-based electronic platform, digital and verifiable vaccination cards were issued to clients recorded in the electronic platform. In the five countries that did not deploy a client-based electronic platform, only paper vaccination cards were available.

## Discussion

The unprecedented rapid progression of the COVID-19 pandemic led to countries quickly developing methods for reporting COVID-19 data, especially the use of digital tools [11]. As COVID-19 challenged health systems around the world, innovators supported governments to develop and adapt digital tools for case management, contact tracing, evidence-based surveillance, risk communication, and vaccine delivery [12]. In this study, 22 (96%) out of the 23 countries that participated in the study deployed digital tools to collect COVID-19 vaccine data and monitor progress in vaccine uptake including 18 countries (78%) with electronic immunization registries. Indeed, given the speed at which the COVID-19 vaccines were delivered and administered from November 2021 onwards, the support of digital technologies played a critical role in facilitating the planning, delivery, monitoring, and management of COVID-19 vaccine programmes [13].

In this study, of the 18 countries that deployed electronic immunization registries for COVID-19, 11 (61%) used the Tracker module of DHIS2, leveraging their past investments in and experience with DHIS2 for routine immunization, disease surveillance, or health management information systems (HMIS). DHIS2 is considered as the world’s largest HMIS platform, used by MoH in 80 low- and middle-income countries [14]. In the African region, 26 countries used DHIS2 for COVID-19 surveillance and vaccine deployment monitoring [14]. These countries leveraged their past experience using electronic platforms instead of developing new solutions. ‘*A crisis is not the time to be trying new things’* is one of the takeaways of the seventh DHIS2 Symposium held virtually in September 2022 [15]. The success of DHIS2 rapid deployment for COVID-19 vaccines has been a combination of a flexible and responsive platform and political commitments to scaling up digital solutions to support efficient, equitable delivery of COVID-19 vaccines, including extensive use of the Tracker module for electronic immunization registries and vaccination certificates [15].

In most countries, not all vaccination sites were using the electronic platform and not all the individual data were transcribed. Key informants identified several reasons contributing to the suboptimal use of digital platforms for decision-making. These reasons include but are not limited to irregular payment of incentives for data clerks, unreliable Internet coverage especially in rural areas, inadequate allocation of Internet subscriptions to vaccination sites, limited proficiency among vaccination teams in data entry, high staff turnover, insufficient supervision, and insufficient availability of electronic devices. According to Mason et al. [12], the sustainability of an electronic platform depends on its local adaptability and usability within the country context. Strengthening local capacity is pivotal in facilitating a successful transition to digital platforms, as it garners greater user acceptance and commitment. Temeslow Mamo [16] from the Tony Blair Institute for Global Change identified three cross-cutting data management challenges currently hindering vaccine rollouts including weak connectivity and infrastructure (lack of data collection equipment and poor connectivity in remote districts were key obstacles), poor data management systems (multiple reporting tools, data not integrated into a single system or platform, lack of interoperability across various systems), and shortage of trained workforce. The suboptimal functioning of electronic immunization registries led to limited access to digital vaccination certificates. In a related study, Mbunge [17] identified the lack of sufficient data as a key impediment encountered by tools that generate COVID-19 electronic vaccination certificates. To ensure the usefulness of a digital system, it is imperative that the data collected be complete and comprehensive. In this study, most countries with less than 100% of individual records entered into the electronic platform had to set up parallel systems for collecting aggregate data for progress monitoring purposes.

To prevent backlogs and errors in data entries, Uganda set up Smart Paper Technology, a digital system that uses paper designed with scannable elements that uploads data to national databases in real time [18]. The use of Smart Paper Technology contributed to clearing an important part of a backlog in individual data entries. As a result, 70% of individual records were transcribed into DHIS2 Tracker despite that only 30% of vaccination sites were using DHIS2. In the context of limited resources and high turnover of health staff, Smart Paper Technology could be regarded as a bridge between paper registers and electronic immunization registries as long as transcription errors are minimized.

Governments in the African region, with partner support, made significant investments in electronic platforms for COVID-19 data management. These investments contributed to better data-driven planning and decision-making [19]. As countries are stepping down their COVID-19 vaccine programmes following the lifting of the public health emergency of international concern status, it is critical to leverage investments made in COVID-19 vaccine data systems. This includes harnessing these resources to strengthen routine immunization data management, especially expanding the deployment and utilization of digital immunization registries. Tools such as the ‘COVID-19 to Routine Immunization Information System Transferability Assessment’ (CRIISTA) under development by John Snow Inc. [20] will help countries optimize COVID-19 vaccine data investments for the future, by developing operational plans for strengthening routine immunization data management.

## Limitations

The assessment of existing data systems for COVID-19 vaccines was conducted in 23 out of the 47 countries in the African region. Countries included in the study were those that responded to the WHO AFRO request for key informants and were not selected randomly. Accordingly, the sample of countries in this study may not be representative of the African region.

Data for this survey were collected through online interviews with key informants. No cross-verification measures were put in place to confirm assertions from the respondents. This could raise concern regarding the quality of data collected during the survey as the key informants might have provided biased responses.

This survey did not deeply examine the quality of the COVID-19 vaccine data recorded using the existing data systems. Future assessments should explore the consistency, timeliness, and completeness of data collected to allow comparability of the different platforms.

The interpretation of the results of this study should take these limitations into account.

## Conclusion

The deployment of COVID-19 vaccines led to unprecedented investments in data systems to monitor progress and inform decisions. Several countries in the African region deployed digital immunization registries using the Tracker module of DHIS2 or locally developed platforms. Unfortunately, challenges related to the availability of required equipment, access to the Internet, and high turnover of trained staff prevent electronic platforms from achieving completeness in data transcribed and therefore from being fully useful for the COVID-19 vaccine programme. Despite these limitations, investments made and the experience acquired in deploying digital platforms for COVID-19 vaccines need to be leveraged to strengthen routine immunization data management. To this end, it is critical for the WHO AFRO and other partners to support countries in assessing the transferability of COVID-19 information systems into routine immunization.

## Data Availability

The data sets generated and/or analysed during the current study are available from the corresponding author upon reasonable request.
